# Nanoliposomal Coencapsulation of *Dorema aucheri* Extract and Curcumin; Enhanced Cytotoxicity, Apoptosis Induction, and Inhibition of EGFR Gene Expression in Oral Cancer Cells OCC-02

**DOI:** 10.1049/2023/1745877

**Published:** 2023-10-26

**Authors:** Mahshid Azizi, Ghasem Ghalamfarsa, Fatemeh Khosravani, Hassan Bardania, Shahriar Azizi

**Affiliations:** ^1^School of Dentistry, Yasuj University of Medical Sciences, Yasuj, Iran; ^2^Medicinal Plants Research Center, Yasuj University of Medical Sciences, Yasuj, Iran; ^3^Student Research Committee, Yasuj University of Medical Sciences, Yasuj, Iran; ^4^Cellular and Molecular Research Center, Yasuj University of Medical Sciences, Yasuj, Iran; ^5^School of Medicine, Yasuj University of Medical Sciences, Yasuj, Iran

## Abstract

Curcumin is one of the natural anticancer drugs but its efficiency is limited by low stability, insufficient bioavailability, poor solubility, and poor permeability. *Dorema aucheri* (Bilhar) is a herb with precious pharmaceutical properties. This study aimed to develop a nanoliposome-based curcumin and Bilhar extract codelivery system. The nanocompounds were synthesized using the lipid thin-film hydration method and characterized by transmission electron microscopy, and dynamic light scattering techniques, and their cytotoxicity and apoptotic effect on the primary oral cancer cell line were evaluated via 2,5-diphenyl-2H-tetrazolium bromide assay and flow cytometry. Moreover, the expression of the epidermal growth factor receptor (EGFR) gene in the treated cells was assessed using the real-time polymerase chain reaction technique. Based on the results, nanoliposomes had a size of 91 ± 10 nm with a polydispersity index of 0.13. Free curcumin, the extract, and the curcumin-extract combination showed dose-dependent toxicity against cancer cells; yet, the extract (IC_50_: 86 *µ*g/ml) and curcumin-extract (IC_50_: 65 *µ*g/ml) activities were much more than curcumin (IC_50_: 121 *µ*g/ml). Also, the curcumin and extract loaded on liposomes showed a dose and time-dependent cytotoxicity. After loading the curcumin-extract compound on nanoliposomes, their IC_50_ decreased from 180 *µ*g/ml (within 24 hr) to 43 *µ*g/ml (within 72 hr), indicating their sustainable release and activity. Likewise, this compound induced the highest apoptosis percentage (95%) in cancerous cells and inhibited the expression of the EGFR gene in the cells by 81% ± 3%. These findings demonstrated the effectiveness of the Bilhar extract against oral cancer cells. Also, in combination with curcumin, it showed an additive activity that considerably improved after loading on nanoliposomes.

## 1. Introduction

Curcumin is a yellow pigment that can mostly be found in turmeric (*Curcuma longa* and *Curcuma aromatica*), plants of the ginger (Zingiberaceae) family, and is well-known as a spice utilized in curry. This polyphenolic compound possesses anti-inflammatory activity and increases the antioxidant levels produced in the body. During the last decade, various studies have demonstrated the potential benefits of curcumin for human health. Scholars have reported biological properties of this compound, such as antioxidant, anti-inflammatory, antiamyloid, antidiabetic, anticystic fibrosis, and antimicrobial properties [1–3]. This phytocompound has gained much attention since its anticarcinogenic activity was revealed. The investigators have designed many studies to investigate the mechanisms of action by which curcumin induces its effectiveness on the disease at various stages [4]. Nevertheless, low aqueous solubility and instability which led to rapid metabolism and clearance are still the drawbacks limiting curcumin nutraceutical and pharmaceutical applications [5]. Various strategies have been developed in recent years to overcome these limitations, such as loading the drug on suitable carriers, chemical modification, and cocrystal synthesis.

Encapsulation of curcumin into lipid-based carriers, such as liposomes, has recently become a very popular and promising approach. In simple terms, liposomes are small vesicles that spontaneously self-assemble when phospholipids are dispersed in an aqueous solution. By forming an internal aqueous core, lipid molecules construct a double-layer structure similar to natural cell membranes, which makes them biocompatible and biodegradable. Furthermore, these features let these nanostructures be broadly used to load both hydrophilic and hydrophobic drugs and overcome cellular and tissue barriers [6].

Considering the side effects and limitations current drugs show, research interests have been turned to natural bioactive products, such as medicinal plants [7–9]. In this regard, *Dorema aucheri* is an herb from the Apiaceae family, which is native to Iran and locally called Bilhar. According to the primary studies, aerial parts of this plant secrete a lipophilic exudate material that contains several flavone methyl esters [10]. Recently, a few studies have investigated the hepatoprotective, antidiabetic, antitumor, antioxidant, antihyperlipidemic, and antihypercholesterolemic activities of this plant extract. Moreover, the beneficial effects of *D. aucheri* extract on thyroid hormones, antioxidant enzymes, and the hematologic system have been reported. Moreover, previous studies have shown the positive effect of this herb consumption on the serum levels of testosterone, follicle-stimulating hormone, and luteinizing hormone [11, 12]. Although the wide spectrum of biological activities of Bilhar has been investigated, no reliable report is available on the anticancer activity of this herb.

Herein, we designed a study to evaluate the anticancer activity of Bilhar extract against oral cancer cells. Furthermore, the activity of this extract in combination with curcumin was assessed. We also loaded these compounds on nanoliposomes in order to take advantage of this drug carrier to enhance the agents' activity. As one of the most promising results, this study revealed that the Bilhar extract-curcumin loaded on nanoliposomes was much more effective against oral cancer cell line (OCC-02) cells in comparison with each of these components alone. This compound showed increased cytotoxic and apoptotic activity while decreasing the expression of the epidermal growth factor receptor (EGFR) gene expression in a dose and time-dependent manner.

## 2. Material and Methods

### 2.1. Extract Preparation

The samples of the aerial part of the Bilhar (*D. aucheri*) were collected from the countryside of Yasuj, Kohgiluyeh, and Boyer-Ahmad province, Iran, in early spring. The samples were used to prepare the Bilhar hydroalcoholic extract, after being authenticated by the Herbarium of Medicinal Plants Research Center, Yasuj University of Medical Sciences (voucher no. 0496). To begin with, the samples were air-dried at the shade (room temperature) and powdered using an electrical mill. Next, 300 g of the powder was immersed in 1,200 ml of ethanol (70%) and shaken for 72 hr at room temperature. The mixture was then filtered using Whatman filter papers (Grade 1), centrifuged (20 min, 3,500 rpm), and the supernatant was dried at 40°C in an oven. The final product was stored at −4°C. And, when needed, the required amount of the extract was weighed and dissolved in methanol (80%) to prepare the intended concentrations [11].

### 2.2. Nanoliposomes and Curcumin/Extract-Loaded Nanoliposomes Synthesis

Thin-layer hydration method was applied to prepare nanoliposomes [13]. Briefly, 30 mg of DPPC (1,2-dipalmitoyl-*sn*-glycerol-3-phosphocholine; Lipoid, Ludwigshafen, Germany) and 10 mg of cholesterol (Sigma–Aldrich, Germany) were separately dissolved in 1 ml of chloroform and the solutions were mixed in a round–bottom flask (2 : 1). Then, the resulting solution was dried under the vacuum in a rotary evaporator at 50°C to evaporate the organic solvent and form a thin lipid layer. Next, the lipid layer was gradually hydrated using phosphate buffer solution for 2 hr. After that, liposomes were sonicated using a water bath ultrasonicator to make unilamellar vesicles.

To synthesize curcumin-loaded nanoliposomes, the procedure mentioned above was followed. The difference is that 5 *µ*l of curcumin solution (5 mg/ml; Arasto Pharmaceutical Chemicals Inc, Tehran, Iran) in chloroform was added to the mixture of DPPC and cholesterol. Likewise, to synthesize the extract-loaded nanoliposomes, 2 mg of the extract solved in 500 *µ*l of methanol was added to the mixture of DPPC and cholesterol. The curcumin (5 *µ*l of curcumin solution 5 mg/ml) and extract (2 mg in 500 *µ*l of methanol) were simultaneously loaded on nanoliposomes using the same method. Centrifugation at 29,000 × *g* for 30 min was conducted to separate nonloaded drugs from liposomes.

### 2.3. Physicochemical Characterization

Transmission electron microscopy (TEM; Philips CM30, Netherlands) was applied to study nanoliposomes' morphology. Also, their average particle size in distilled water was determined by dynamic light scattering (DLS) analysis (Malvern, Zetasizer Nano ZS Instruments) using an argon laser beam at 633 nm and a scattering angle of 90 at room temperature (25°C). Also, the histogram of particle size was determined by analyzing the TEM results with ImageJ software. Measurements were performed in triplicate.

### 2.4. Cell Culture

To evaluate the cytotoxicity of the prepared compounds, the primary OCC-02 was purchased from the Iranian Biological Resource Center, Tehran, Iran. A humid environment at 37°C and 5% CO_2_ was used to culture the cells in Roswell Park Memorial Institute medium with 10% fetal bovine serum and 10% penicillin–streptomycin (Gibco-Thermo Fisher Scientific, USA).

### 2.5. MTT Assay

The cytotoxic activity of nanoliposomes, curcumin, curcumin-loaded nanoliposomes, Bilhar extract, the extract-loaded nanoliposomes, and nanoliposomes loaded with curcumin-extract against OCC-02 cells was studied using 2,5-diphenyl-2H-tetrazolium bromide (MTT) assay [14]. In this experiment, curcumin was dissolved in dimethyl sulfoxide (DMSO; 0.2%), the extract was dissolved in methanol and the extract and curcumin were mixed with a ratio of 1 : 1 (w/w). To begin, 7 × 10^3^ cells were seeded in 96-well plates and incubated for 24 hr. Next, the cells were treated with different concentrations of each agent (31.25, 62.5, 125, and 250 *μ*g/ml) for 24, 48, and 72 hr. After the respective incubation period, the contents of the wells were drained, and 100 *μ*l of MTT solution (5 mg/ml; Sigma–Aldrich, Germany) was added to each one, followed by incubating the plates for 4 hr. After that, the contents of the wells were replaced with 100 *μ*l of DMSO solution, and the plates were incubated for 40 min. Finally, the absorbance of the wells was measured using an ELX800 UV universal microplate reader (Bio-Tec Instruments Inc., Vermont, USA) at 570 nm. In this experiment, nontreated cells were considered as the control. Cell viability was calculated using the following equation.

(1) $$\begin{array}{c} \text{Cell viability }\left(\%\right)=\left(\frac{\text{Absorbance of sample wells}}{\text{Absorbance of control wells}}\right)\times 100. \end{array}$$

The combination index (CI) for the curcumin and the extract was calculated following the Chou–Talalay method [15]. In this calculation, we used the compounds IC_50_ values obtained after 48 hr of treatment. The CI was calculated using Equation (2).

(2) $$\begin{array}{c} \text{CI }=\left(\frac{{\text{IC}}_{50}\text{ of curcumin in combination}}{{\text{IC}}_{50}\text{ of curcumin alone}}\right)+\left(\frac{{\text{IC}}_{50}\text{ of the extract in combination}}{{\text{IC}}_{50}\text{ of the extract alone}}\right). \end{array}$$

### 2.6. Apoptosis Assay (Flow Cytometry)

The apoptotic cells exposed to the compounds were determined by flow cytometry analysis using the Annexin V/PI apoptosis kit (United Biotechnology Co., Ltd., Shanghai, China) following the instructions provided by the manufacturer. Briefly, the cells were seeded in a 6-well plate and incubated for 24 hr. Next, they were treated with the compounds at their IC_50_ concentrations and incubated for 24 hr. After trypsinizing and washing the cells with binding buffer, 5 ml of Annexin V and 5 ml of PI were added to the cells along with 150 ml of binding buffer. Finally, a Becton Dickinson FACS Calibur flow cytometer was used to analyze the cells after they were kept in the dark for 20 min.

### 2.7. EGFR Gene Expression Assessment

The expression of the EGFR gene in the cells exposed to the compounds was assessed using the real-time polymerase chain reaction (PCR) technique. Toward this end, the RNX-Plus reagent (CinnaGen Co., Iran) was applied to extract total RNA from the cells. A spectrophotometer (NanoDrop™ 2000/2000c) was used to analyze the purified RNA after dissolving it in 30 *µ*l of diethyl pyrocarbonate-treated water. By loading the products on agarose gel electrophoresis (1.5%; Sigma, Taufkirchen, Germany), the integrity of the extracted RNA was also confirmed. Afterward, the Takara cDNA synthesis kit was utilized to synthesize cDNA, in accordance with the instructions provided by the manufacturer.

The cDNA obtained from each sample was used to assess the mRNA expression of the EGFR gene using a BIO-RAD CFX96 detection system (Bio-Rad, Richmond, USA). This was carried out using the 2x EmeraldAmp GT PCR Master Mix (Takara Bio Inc., USA). And, to normalize the expression of target genes, the Glyceraldehyde 3-phosphate dehydrogenase gene (*gapdh*) was used as an internal control. The following real-time PCR program was applied in this study; initial denaturation for 15 min at 95°C, followed by 40 cycles for real-time PCR, including denaturation phase: 20 s at 95°C, annealing phase: 30 s at 56°C, and elongation phase: 40 s at 72°C. To check for primer dimers and product specificity, a final melting curve was conducted. A 1.5% agarose gel electrophoresis was also performed to confirm the products of each primer pair.  Table 1 shows details about the primer sequences. It is worth mentioning that each sample was evaluated in triplicate, and the means of the samples were considered in further evaluations. The relative levels of mRNA expression were determined based on the *ΔΔ*CT method.

### 2.8. Statistical Analyses

All experiments were repeated three times, and the results were presented as mean ± standard deviation. One-way analysis of variance with Tukey's post hoc test was used to analyze data and determine whether the differences were statistically significant.

## 3. Results

### 3.1. Nanoliposomes' Characteristics

**Figure 1:**
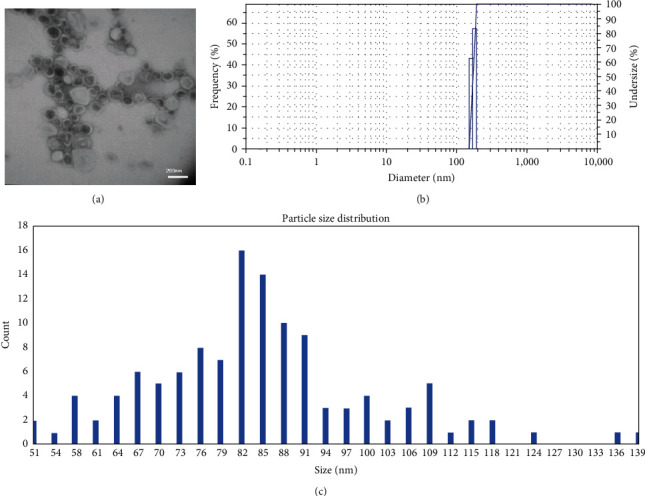
The results of TEM (a) and DLS (b) analysis of the nanoliposomes synthesized using the thin film method. (c) Shows the particle size distribution obtained from analyzing the TEM image using ImageJ software.

Having nanoparticles synthesized, their morphological features were studied using TEM microscopy and DLS analysis. As results showed (Figure 1), the synthesized nanoliposomes were spherical with 91 ± 10 nm diameter in size (polydispersity index = 0.132). Both characterization techniques' results confirmed the monodispersity of the nanoliposomes, demonstrating the success of the synthesis process.

### 3.2. Compounds' Cytotoxicity

**Figure 2:**
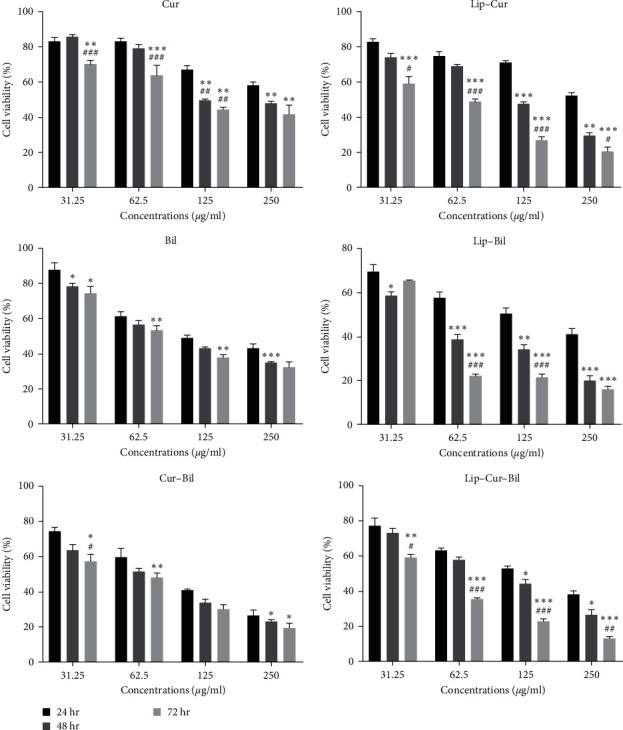
The viability of the oral cancer cells exposed to the different concentrations of curcumin (Cur), curcumin-loaded liposomes (Lip–Cur), Bilhar extract (Bil), the extract-loaded liposomes (Lip–Bil), curcumin combined with the extract (Cur–Bil), and liposomes loaded with the extract and curcumin (Lip–Cur–Bil). Here,  ^*∗*^indicates significant differences with 24 hr at  ^*∗*^*P* < 0.05,  ^*∗∗*^*P* < 0.01,  ^*∗∗∗*^*P* < 0.001. And ^#^indicates significant differences with 24 hr at ^#^*P* < 0.05, ^##^*P* < 0.01, ^###^*P* < 0.001.

The cytotoxicity of the compounds toward cancerous cells was evaluated using the MTT assay. As results showed, all compounds had activity against cancerous cells. However, the composition of curcumin-extract loaded on nanoliposomes was considerably more effective on the cells. Also, they showed a time and dose-dependent activity, while free drug and extract activity were not time-dependent significantly (Figure 2).

Concerning the free curcumin, the average cell viability, after 24, 48, and 72 hr of treatment, was about 50% (at 250 *µ*g/ml). Yet, at the same concentration and periods, the viability of the cells treated with liposome-curcumin reduced to 52%, 29%, and 20%, respectively.

Furthermore, the free Bilhar extract could decrease the viability of the cells by about 65% during 72 hr. Similar to liposome-curcumin, the liposome-extract compound showed considerable time-dependent activity. After 24 hr of treatment, 250 *µ*g/ml of this agent resulted in 41% cell viability, while this feature reduced to 15% after 72 hr.

As our results revealed, the cotreatment of the curcumin and the extract presented superior anticancer activity. This combined agent could reduce cell viability at markedly lower concentrations. Based on our calculations, the CI obtained for the compounds was 1.1 ± 0.13, indicating the additive effect of the combination. Also, the extract-curcumin-loaded liposomes showed the most desirable activity. From 24 to 72 hr of treatment, the survival percentage of the cells exposed to this compound from 38% considerably decreased to 12%.

**Figure 3:**
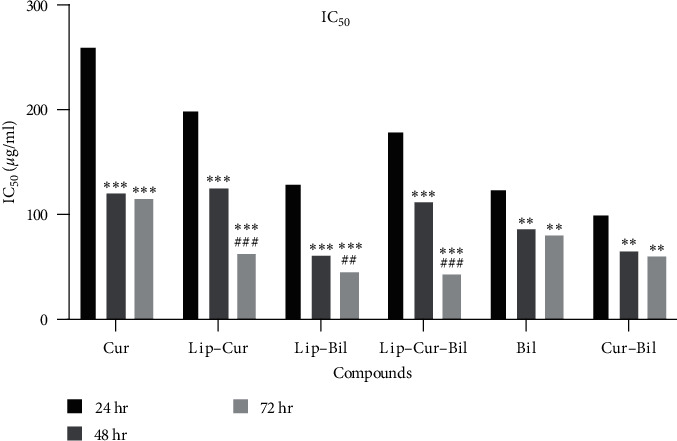
IC_50_ values of curcumin (Cur), curcumin-loaded liposomes (Lip–Cur), Bilhar extract (Bil), the extract-loaded liposomes (Lip–Bil), curcumin combined with the extract (Cur–Bil), and liposomes loaded with the extract and curcumin (Lip–Cur–Bil) against OCC-02 cells. Here,  ^*∗*^indicates significant differences with 24 hr at  ^*∗*^*P* < 0.05,  ^*∗∗*^*P* < 0.01,  ^*∗∗∗*^*P* < 0.001. ^#^Indicates significant differences with 24 hr at ^##^*P* < 0.01, ^###^*P* < 0.001.

Figure 3 compares the IC_50_ values of the compounds. Based on the results, the liposomes containing curcumin and Bilhar extract with the lowest IC_50_ and the most difference between 24 and 72 hr of treatment showed the best performance. The IC_50_ of this compound was 181 *µ*g/ml at 24 hr, which decreased to 43 *µ*g/ml after 72 hr.

### 3.3. Apoptosis Assay

**Figure 4:**
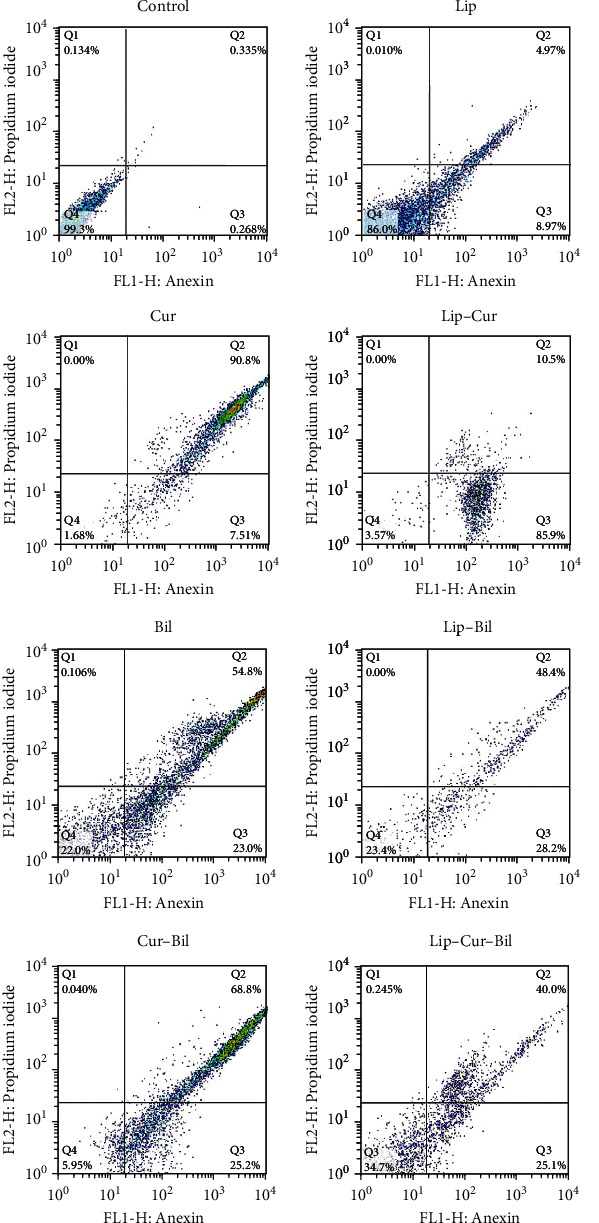
The flow cytometry analysis of the apoptosis incidence in the cells treated with curcumin (Cur), curcumin-loaded liposomes (Lip–Cur), Bilhar extract (Bil), the extract-loaded liposomes (Lip–Bil), curcumin combined with the extract (Cur–Bil), and liposomes loaded with the extract and curcumin (Lip–Cur–Bil).

**Figure 5:**
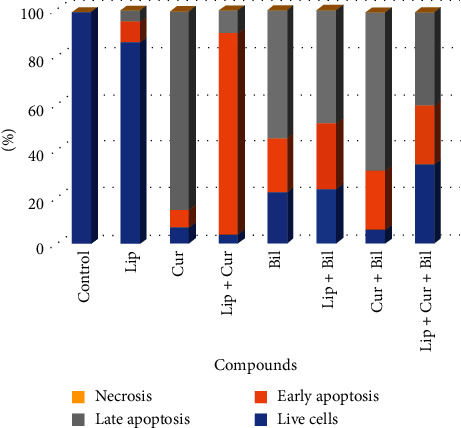
The percentage of the apoptosis incidence in cells based on the flow cytometry analysis. Cur, curcumin; Lip–Cur, curcumin-loaded liposomes; Bil, Bilhar extract; Lip–Bil, the extract-loaded liposomes; Cur–Bil, curcumin combined with the extract; Lip–Cur–Bil, liposomes loaded with the extract and curcumin.

Flow cytometry analysis was done to determine the apoptotic cells exposed to the compounds. As our results showed, all compounds could induce apoptosis in the treated cells (Figures 4 and 5). Curcumin caused about 97% apoptosis incidence (7% early and 90% late apoptosis) in the cells. Also, regarding the liposomes-curcumin compound, this feature was 96% (86% early and 10% late apoptosis). Moreover, the apoptosis induced by the Bilhar extract was more than 77%. A similar apoptosis rate was observed in the cells treated with the extract-loaded liposomes. Concerning the cells treated with the combination of curcumin and extract, 95% of them were apoptotic. Not surprisingly, the rate of apoptosis in the liposomes-curcumin-extract treatment was lower (65%) than in free agents. This lower activity is related to the sustainable and controlled release property of liposomes. No marked necrosis was determined in the cells.

### 3.4. EGFR Gene Expression

**Figure 6:**
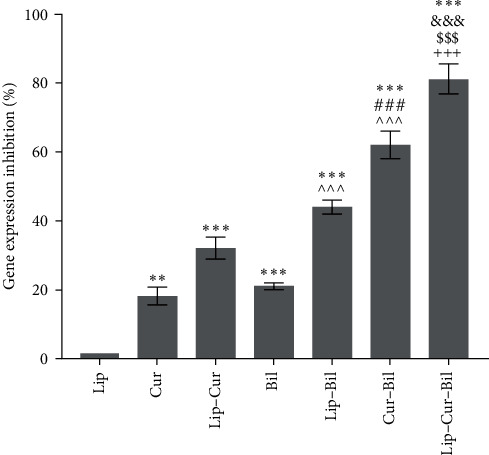
The percentage of EGFR gene expression inhibition in the OCC-02 cells exposed to the curcumin (Cur), curcumin-loaded liposomes (Lip–Cur), Bilhar extract (Bil), the extract-loaded liposomes (Lip–Bil), curcumin combined with the extract (Cur–Bil), and liposomes loaded with the extract and curcumin (Lip–Cur–Bil).  ^*∗*^ and  ^*∗∗*^ shows significant differences with Lip (*P* < 0.05 and *P* < 0.001). ^###^Indicates significant differences with Cur (*P* < 0.001). ^^^^^Indicates significant differences with Bil (*P* < 0.001). ^&&&^Indicates significant differences with Lip–Cur (*P* < 0.001). ^$$$^Indicates significant differences with Lip–Bil (*P* < 0.001). ^+++^Indicates significant differences with Cur–Bil (*P* < 0.001).

Figure 6 shows the results of the real-time PCR technique. Treatment of the cells with the compounds inhibited the expression of the EGFR gene. Among these compounds, liposomes containing curcumin and Bilhar extract with 85% and free curcumin with about 20% gene expression inhibition were the most and the least effective compounds, respectively. Noteworthy, the combination of curcumin and extract, as the second most effective compound, inhibited the expression of the gene well above 60%.

## 4. Discussion

In the present study, we investigated the anticancer activity of free Bilhar extract and the extract loaded on nanoliposomes against oral cancer cells (OCC-02). We also evaluated the activity of the combination of the extract with curcumin. As our results revealed, the extract alone could effectively kill the cancerous cells through apoptosis induction and inhibition of the EGFR gene expression. However, its combination with curcumin had a much better performance. Furthermore, it was revealed that loading these agents on nanoliposomes considerably enhanced their efficiency.

The anticancer properties of curcumin have been proven by researchers already. Our study showed this drug suppressed the EGFR gene expression. The main mechanisms by which curcumin plays its anticancer role are inducing apoptosis and inhibiting proliferation and invasion of tumors by suppressing a variety of cellular signaling pathways. However, investigations have been focused on overcoming the pharmaceutical limitation of this drug by taking advantage of drug delivery systems. Based on our results, nanoliposomes could efficiently carry this drug and enhance its cytotoxic activity. This improved activity is associated with the point that nanoliposomes can increase curcumin water solubility and facilitate its uptake by cells [16, 17].

As our study revealed, the Bilhar extract has considerable activity against primary oral squamous cell carcinomas. Similar activity has also been reported against MCF7, HepG2, MDBK, and A549 cells already [18]. This activity can be attributed to the phytochemicals present in the extract. Based on the literature, the aerial parts of *D. aucheri* are rich in flavonoids, a large group of polyphenolic compounds [11]. There is solid evidence indicating the anticancer activity of these phytocompounds [19–21]. Also, in agreement with previous investigations, loading the extract on nanoliposomes caused an enhanced and time-dependent cytotoxicity. The drugs' controlled release is of imperative features made possible by using nanoliposomes as drug carriers. These lipid-based nanostructures provide the sustainable release of drugs and prolong their effectiveness time [22, 23].

Our study revealed that the combination of Bilhar extract with curcumin exhibited an additive cytotoxic effect. This finding holds promise as it suggests that utilizing a combination therapy approach could allow for the administration of lower concentrations of each component. Consequently, this has the potential to minimize the occurrence of drug-related side effects, enhancing the overall safety profile of the treatment strategy [24]. Although no precise mechanism of action can be provided for this addictive activity, it could be proposed that by coadministration of curcumin and the extract, the pathways involved in apoptosis induction are expanded.

Curcumin has been shown to possess both pro-oxidant and antioxidant properties, depending on the cellular context and concentration. At low concentrations, curcumin acts as an antioxidant by scavenging reactive oxygen species (ROS) and inhibiting oxidative stress. It can directly neutralize free radicals and prevent oxidative damage to cellular components [25]. However, at higher concentrations or under certain conditions, curcumin can induce oxidative stress by increasing ROS levels. It activates redox reactions within cells which subsequently trigger the production of ROS and increase the expression of apoptosis receptors on the cancerous cell membrane [26]. Also, this drug induces the expression and activity of p53, which causes cell growth inhibition and apoptosis induction. In addition, the potent inhibitory effect of curcumin on the activity of NF-*κ*B and COX-2, which play a role in the overexpression of antiapoptosis genes, such as Bcl-2, has been determined. Besides, it has been revealed that curcumin down-regulates the PI3K antiapoptotic protein while increasing the expression of MAPKs to induce intracellular ROS production and subsequent cell death [27].

Our study determined that curcumin, the Bilhar extract, and their combination could decrease the expression of the EGFR gene and increase the apoptosis rate in cancerous cells. This protein, from the ErbB family, is a tyrosine kinase receptor involved in numerous processes affecting tumor growth, progression, differentiation, invasion, metastasis, and apoptosis inhibition [28, 29]. Recent studies have also demonstrated the noticeable overexpression of EGFR in oral cancer cells [30]. It seems that the overexpression of EGFR in OCCs depends on the tumor stage, invasion, metastasis to the lymph nodes, distant metastasis, and differentiation of cancer [28, 31]. Considering the epithelial origin of most cancers, EGFR expression deserves to be considered in the cancer treatment processes. Fortunately, our findings demonstrated the high ability of the curcumin, the Bilhar extract, and their combination to inhibit the expression of this gene and induce apoptosis in cancer cells.

In conclusion, Bilhar (*D. aucheri*) is a herb from the Apiaceae family and native to Iran. This plant extract showed a potent effect against oral cancer cells. In addition, it showed additive and sustainable activity when codelivered with curcumin via nanoliposomes. Encapsulation of these agents with nanoliposomes improves their water solubility and bioavailability. These agents could also inhibit the EGFR gene expression, induce apoptosis, and kill cancerous cells. Providing a clear mechanism for this additive activity needs further studies. However, our findings highlight the promising anticancer activity of the Bilhar extract, and its additive cytotoxicity in combination with curcumin, especially when encapsulated in nanoliposomes.

## Figures and Tables

**Table 1:** Details of materials used in the RT-PCR procedure.

Primer name	Primer sequence	PCR product size	NCBI accession number
GAPDH	F: TGCACCACCAACTGCTTAGC R: GCAGGGATGATGGTTCTGGAG	174	NM_001357943.2

EGFR	F: 5′-TGCGTCTCTTGCCGGAAT-3′ R: 5′-GGCTCACCCTCCAGAAGGTT-3′	71	XM_047419953.1

## Data Availability

Data sharing is not applicable to this article as no new data were created or analyzed in this study.
